# The relationship between physical performance measures, bone mineral density, falls, and the risk of peripheral fracture: a cross-sectional analysis

**DOI:** 10.1186/1471-2458-9-297

**Published:** 2009-08-18

**Authors:** Hamza Khazzani, Fadoua Allali, Loubna Bennani, Linda Ichchou, Laila El Mansouri, Fatima E Abourazzak, Redouane Abouqal, Najia Hajjaj-Hassouni

**Affiliations:** 1 Department of Rheumatology, El Ayachi hospital, University Hospital of Rabat-Sale, Sale, Morocco; 2 Laboratory of Information and Research on Bone Diseases (LIRPOS), Faculty of Medicine and Pharmacy, Rabat, Morocco; 3 Laboratory of Biostatistics, Clinical Research and Epidemiology (LBRCE), Faculty of Medicine and Pharmacy, Rabat, Morocco

## Abstract

**Background:**

Several factors, in addition to low bone mineral density (BMD), have been identified as risks for fractures, including reduced levels of physical activity, poor balance and low physical performance. The aim of this study was to evaluate the relationship between physical performance measures, BMD, falls, and the risk of peripheral fracture in a population sample of Moroccan women.

**Methods:**

484 healthy women were included. Three measures were used to assess physical performance: timed get-up-and-go test 'TGUGT', five-times-sit-to-stand test '5 TSTS' and 8-feet timed walk '8 FTW'. The association between physical performance measures and BMD, peripheral fracture and falls was performed by univariate and multivariate analysis.

**Results:**

The mean age was 55.1 years. Higher 'TGUGT', '5 TSTS', '8 FTW' test scores were associated with lower BMD measured at different sites (p range from < 0.001 to 0.005). The relationship between the three tests and BMD in all measured sites remained significant after multiple linear regression (p range from <0.001 to 0.026). In the group of post-menopausal patients, the scores of 'TGUGT' and '8 FTW' were significantly higher in fractured patients compared with patients without. After logistic regression, a score of 'TGUGT' > 14.2 sec, a score of '5 TSTS' > 12.9 sec and a score of '8 FTW' > 4.6 sec respectively, increased the probability of anterior peripheral fracture by 2.7, 2.2 and 2.3 (OR = 2.7; 95% CI = 1.2–6.4, OR = 2.2; 95% CI = 1.1–5.2; and OR = 2.3; 95% CI = 1.1–5.1). There was a significant positive correlation between the number of fall/year and the 3 tests. This correlation persisted after poisson regression.

**Conclusion:**

This study suggested that low physical performance is associated with low BMD, and a high risk of history of falls and fractures.

## Background

Osteoporosis is a major public health problem. There are an estimated 1.5 million fragility fractures in the United States each year, including 700,000 spine fractures, 300,000 hip fractures, and 250,000 wrist fractures [1]. Approximately 50% of patients who sustain a hip fracture lose the ability to walk independently; up to 24% of women and 30% of men die within the first year [2,3].

In current clinical practice, most clinicians dealing with established vertebral osteoporosis focus their attentions on bone mineral density (BMD) and rarely consider fall prediction or prevention. Indeed, the risk of fracture is influenced by both bone strength and falls. Measures of physical function and performance are predictors of falls, and both BMD and physical performance are independent predictors of fracture risk [4,5].

Balance impairment worsens with age and has been identified as a risk factor of fractures [6]. Physical training improving muscular strength and leading to a better balance control might decrease the incidence of falls [7]. Indeed, patients with strong leg muscles have a better balance control than those with weaker leg muscles. This has been proved in nursing homes residents among older people with a history of falls, compared with age-matched controls [8].

Many balance tests have been shown to predict future falls in older people [9]. These include the following simple tests, which may be used in a busy clinical setting: the 'timed get-up and go test', the 'times-sit-to-stand test' and the 'gait speed test'.

The aim of the study was to evaluate the relationship between physical performance measures, BMD, falls, and the risk of peripheral fracture in a population sample of Moroccan women.

## Methods

### Subjects

484 healthy Moroccan volunteer women were recruited from the city of Rabat, through advertisements in local hospitals. Patients were referred to our outpatient Bone Densitometry Center from June to August 2006. The mean age of the patients was 55.1 ± 9.6 years. Informed consent was obtained from all patients and the study was approved by the ethics committee of our university hospital. We excluded patients (30% of people who volunteered for the study) with a history of (1) using medications known to influence bone metabolism within the past two years (e.g. vitamin D, calcium, corticosteroids, bisphosphonates and hormone replacement therapy); (2) musclo-skeletal, thyroid, parathyroid, adrenal, hepatic, or renal disease; (3) malignancy; or (4) hysterectomy.

### Data Collection and Measurements

Each patient completed a questionnaire to assess demographic characteristics and osteoporosis risk factors. We also collected data relating to the personal history of peripheral osteoporosis fractures (including proximal femoral fractures) and the self-report history of falls occurring in the last year (a fall defined as any event that led to an unplanned, unexpected contact with a supporting surface).

### Anthropometric Data

Weight and height were measured without clothes or shoes at the time of bone densitometry measurements. The body mass index (BMI) was calculated as body weight (kg)/height (m2).

### Physical Performance Measures

Three measures were used to assess physical and balance performance: timed get up and go test 'TGUGT', five-times-sit-to-stand test '5 TSTS' and 8-feet timed walk '8 FTW'. Time was measured by stopwatch and rounded to the nearest hundredth of a second.

Timed Get Up and Go Test: In this test, the patient rises from a chair, walks 3 meters, turns around, returns to the chair, and sits down [10]. The time taken to complete the task was the score. The Timed Get Up and Go Test was used to evaluate the functional mobility of the participants. Several studies reported high test-retest reliability [11] (ICC = 0.97) and excellent intra- and inter-reliability [10] (ICC = 0.99) for the Timed Get Up and Go test.

Five-times-sit-to-stand test: The sit-to-stand test is commonly used to assess lower extremity strength and balance [12]. The subjects began by crossing their arms on their chest and sitting with their back against the chair (45 cm higher from the floor). Participants were asked to stand up and sit down five times as quickly as possible and were timed from their initial sitting position to the final standing position at the end of the fifth stand. The subjects were reminded to straighten their legs fully when standing. In previous studies with measurements of the same test situation, the sit-to-stand test has shown high reliability [13].

8-feet (2.4 m) timed walk: Patients were instructed to walk as fast as possible for 8 feet (2.4 m). Patients wore the footwear they normally used. A digital stopwatch was used to measure the time between the start of walking and when the first foot crossed the finish line. The reliability of this protocol is reported as adequate [14]. Measurement of gait speed for a short distance is used both clinically and in large epidemiological studies, such as established populations for epidemiological studies of older subjects (2.4 m [8 ft]). Gait speed has been associated with activity level [15] changes in the isometric force of lower extremity muscles [15], self-rated health, and falls [16].

### Dietary Calcium Questionnaire

Dietary calcium intake was assessed with the frequential self-questionnaire of Fardellone [17]. This questionnaire has been modified, simplified and adjusted to the food habits of Moroccans. After translation and back translation, it was administered to 62 volunteers women, aged between 30 and 60 years. To test its validity, the questionnaire was compared to the weekly docket system, chosen as a reference method. To test its reproducibility, the questionnaire was re-administered after one week to the same sample. The coefficient of correlation was 0.91. The questionnaire correctly classified women with daily calcium intake less than 800 mg with 76.9% specificity, while its sensitivity was 86.7%.

### Physical Activity

For the evaluation of physical activity, we used the short form of the International Physical Activity Questionnaire (IPAQ) [18]. The items of IPAQ were structured to provide separate scores on walking, moderate-intensity and vigorous-intensity activity. Computation of the total score requires summation of the duration (in minutes) and frequency (days) of walking, moderate-intensity and vigorous-intensity activities.

### Bone mineral density (BMD) measurements

Lumbar spine, trochanter, femoral neck and total hip BMD were measured by DXA (Lunar Prodigy densitometer). Daily quality control was performed using Lunar Phantom measurements, which showed stable results during the study. The Lunar Phantom showed a precision of 0.08 expressed as the coefficient of variation (CV) in percent. Both T and Z scores were obtained. T-scores were calculated using the manufacturer's European reference population range because no Moroccan reference ranges were available.

### Statistical Analysis

All analyses were performed using SPSS, version 10.0 for Windows (SPSS Inc., Chicago, IL, USA). Results with p values less than 0.05 were considered statistically significant.

Results for continuous variables are expressed as mean ± standard deviations. Comparison was made by the Student t-test. Categorial variables were compared by using the chi-square test.

Using the receiver operating characteristic (ROC) curve, we determined the best cut-off point for each physical performance measure to discriminate osteoporosis patient. The best cut-off values have been chosen according to the best sensitivity and specificity (closest to the left upper corner of the ROC curve). The subjects were separated into 2 groups (group 1 was below and group 2 was above the best cut-off point) to conclude whether subjects who displayed better physical performance also had higher BMD.

We conducted univariate analysis to identify clinical variables significantly associated with BMD, with falls and with history of peripheral fracture: age, BMI, age of menarche, age at menopause, number of pregnancies, total calcium intake and current hours of total physical activity. Next, multivariate analyses were performed using: multiple linear regression for BMD, logistic regression for peripheral fracture and poisson regression for the number of falls. The covariates with a p value < 0.10 in univariate analysis (age, BMI, age of menarche, total calcium intake and current hours of total physical activity) were included in 3 models of multivariate analyses and we performed a stepwise forward selection procedure.

## Results

### Clinical characteristics

The characteristics of subjects are shown in Table 1. The mean BMI was 28.2 ± 4.7 kg/m^2^. Of the 484 participants, 175 (31.2%) reported a history of falling. Among menopausal women (n = 360), 31% were osteoporotic at any of the measured sites (spine, hip) (we used the WHO classification of osteoporosis which defined osteoporosis: BMD 2.5 SD or more below the young adult mean [T-score at or below -2.5]) and 11.9% had a personal medical history of peripheral fractures (including proximal femoral fractures). The mean daily dietary calcium intake was 694 ± 231 (range 190 to 1800) mg and the median total physical activity was 2346 minutes/week (interquartile range, 929–4918). The best cut-off point for physical performance measures was: 14.2 sec for 'TGUGT', 12.9 sec for '5 TSTS' and 4.6 sec for '8 FTW'.

**Table 1:** Clinical and osteodensitometric characteristics for the studied population

	**Mean ± SD**		
*Age (y)*	55.1 ± 9.6		
*Age of onset of menarche (y)*	12.7 ± 1.8		
*Weight (kg)*	71.2 ± 11.5		
*Height (cm)*	156.9 ± 8.8		
*BMI (kg/m2)*	28.2 ± 4.7		
*Total physical activity (min/wk)*	2346 ± 1017		
*Total calcium intake (mg/d)*	694 ± 231		
***BMD (g/cm2)***			
*Lumbar spine*	1.011 ± 0.180		
*Trochanter*	0.720 ± 0.127		
*Femoral neck*	0.881 ± 0.151		
*Ward's triangle*	0.729 ± 0.161		
*Total hip*	0.925 ± 0.151		

*T-score Lumbar*	**Median**	**min**	**max**
	-1.6	-5.4	2.5
*T-score Hip*	-0.9	-3.5	2.3

Abbreviations: "wk": week;"d": day.

### The Relationship between Physical Performance Tests and BMD

In univariate analyses, higher 'TGUGT', '5 TSTS', '8 FTW' test scores were associated with lower BMD measures at different sites (r range from -0.20 to -0.13; p range from < 0.001 to 0.005). These associations were weak but statistically significant.

**Figure 1:**
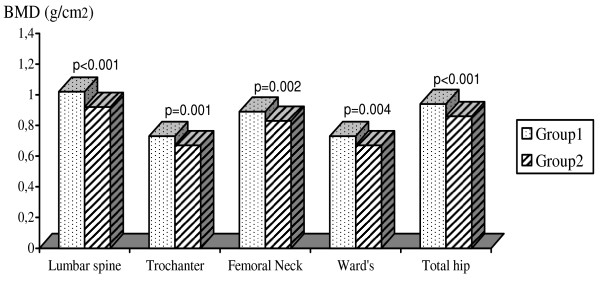
**Means for BMD of various skeletal sites according to the timed get-up-and-go test (TGUGT)**. Abbreviations: BMD, bone mineral density. Group 1 was below the best cut-off (≤ 14.25 sec) and group 2 was above the best cut-off (>14.25 sec).

When subjects were divided into below the best cut-off (group 1) and above the best cut-off (group 2) for '5 TSTS', '8 FTW' and 'TGUGT' (Figure 1), those in group 1 had significantly higher BMD in all measured sites.

The relationship between the three tests and BMD in all measured sites remained significant after adjustment for BMI, age of menarche, total calcium intake and hours of total activity (p range from < 0.001 to 0.026) (Table 2).

**Table 2:** The relationship between the three tests and BMD after multiple regression models

BMD	Spine		Femoral Neck		Trochanter		Total hip	
	β	p	β	p	β	p	β	p
"TGUGT"	-7.33^-03^	< 0.001	-5.26^-03^	0.001	-4.00^-03^	0.001	-7.02^-03^	< 0.001
"5 TSTS"	-4.82^-03^	0.010	-3.65^-03^	0.026	-4.85^-03^	< 0.001	-4.52^-03^	0.004
"8 FTW"	-1.37^-02^	0.002	-1.15^-02^	0.003	-8.79^-03^	0.004	-1.93^-02^	< 0.001

Abbreviations: "TGUGT": Timed get-up-and-go test; "5 TSTS": Five-times-sit-to-stand test; "8 FTW": 8-feet timed walk; "BMD": bone mineral density.

Each physical performance measure was entered separately into the model because of colinearity. Models were corrected for BMI, age of onset of menarche, total calcium intake, and total minutes of physical activity.

### The Relationship between Physical Performance Tests and Peripheral Fracture

**Figure 2:**
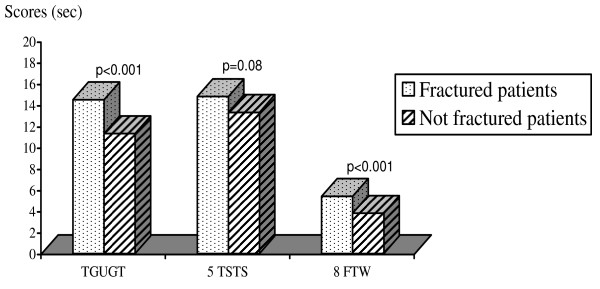
**Comparison of the scores of the 3 tests between the fractured vs not fractured patients**. Abbreviations: TGUGT, Timed get-up-and-go test; 5 TSTS, Five-times-sit-to-stand test; 8 FTW, 8-feet timed walk; sec, second.

In the sub-group of post-menopausal patients, the scores of the tests 'TGUGT', and '8 FTW' were significantly higher in fractured patients compared with women with no previous fractures (14.5 sec ± 8.2 sec vs 11.4 sec ± 4.8 sec; p < 0.001 and 5.4 sec ± 2.6 sec vs 3.9 sec ± 2.0 sec; p < 0.001 respectively); while test '5 TSTS' scores were approaching significance (14.8 sec ± 6.4 sec vs 13.3 sec ± 5.1 sec; p = 0.08) (Figure 2).

After adjusting for age, BMI and total hip BMD by logistic regression, a score of 'TGUGT' > 14.2 sec, a score of '5 TSTS' > 12.9 sec and a score of '8 FTW' > 4.6 sec respectively, increased the probability of anterior peripheral fracture by 2.7, 2.2 and 2.3 (OR = 2.7; 95% confidence interval (CI) = 1.2–6.4; p = 0.019, OR = 2.2; 95% CI = 1.1–5.2; p = 0.049 and OR = 2.3; 95% CI = 1.1–5.1; p = 0.033).

### The Relationship between Physical Performance Tests and Falls

Performance in the three tests was significantly worse in the group with a history of falls, as compared with the group without. These differences were statistically significant (p = 0.003 for 'TGUGT', p < 0.001 for '5 TSTS' and p < 0.001 for '8 FTW'). Furthermore, there was a significant positive correlation between the number of falls/year and the 3 tests. This correlation persisted after adjusting for age (Table 3).

**Table 3:** Relation between the number of fall/year and the three tests

	*Risk Ratio*	*CI 95%*	*p*
*TGUGT (per sec)*	1.03	1.01 – 1.05	0.021
*5 TSTS (per sec)*	1.04	1.02 – 1.07	0.001
*8 FTW (per sec)*	1.13	1.07 – 1.18	< 0.001

Abbreviations: "TGUGT": Timed get-up-and-go test; "5 TSTS": Five-times-sit-to-stand test; "8 FTW": 8-feet timed walk.

This relation was considered after adjusting for age by poisson regression method.

## Discussion

In this population-based study, we showed that low physical performance is associated with reduced BMD at both the spine and hip in women. All of the measures, showed consistent significant associations with hip and lumbar spine BMD in simple correlations and multiple regression models, that were controlled for confounders already known to influence BMD. Our results are consistent with the majority of previous studies among women, showing an association between physical performance and BMD at the spine and the hip [19-21].

Taaffe et al [22] found that physical capacity assessed by repeated chair stands, gait speed, walking endurance, and standing balance was only modestly related to BMD at the hip. In another study, Lindsey et al [21] showed that physical performance was associated with hip, spine and whole body BMD, using normal and brisk gait speeds, normal and brisk step length and one leg stance time. Several studies validate repeated sit to stand time as a measure of lower-extremity strength and power [12,23], and quadriceps strength has been associated with femoral neck BMD in similar samples [24].

Physical performance is reflected in lower extremity strength and gait speed. Activity produces a mechanical load on the bone through muscle contraction and surface impact, which contributes to bone formation and remodeling. It is considered that a lack of physical activity reduces mechanical load on bones, which can then lead to a decrease in bone density. The positive effect of walking speed on hip and lumbar spine BMD is in line with interventional exercise studies showing that regular weight bearing and/or resistance exercise over extended time periods [21] could maintain or slightly increase hip and lumbar spine BMD in older women. In light of evidence that even the force of walking can cause a femoral neck fracture when BMD is very low, it stands to reason that increased force generated by walking would stimulate bone formation at that site [21].

In post-menopausal patients, we have found that women with self-reported prior fractures have inferior performance scores for 'TGUGT', '5 TSTS' and '8 FTW' compared with women with no previous fractures. This result agrees with Gerdhem, [25] who found that previous fractures are associated with inferior physical performance (Romberg test and gait speed test) in older women. This is also consistent with the prospective study on the effect of fracture on physical performance. In a longitudinal case-cohort, Greendale et al [26] reported that individuals with a hip, arm, or clinical spinal fracture show global declines in physical performance compared with individuals without fractures. In retrospective studies there is always the question of what comes first: impaired balance leading to a fracture? Or fracture leading to impaired balance?

Inferior balance capability is associated with the tendency to fall, which is one of the more important risk factors for fractures [27], and interventions for fall prevention include balance training. Additionally, a possible cause for a slower physical performance in the "fractured" group might be atrophy due to prolonged bed rest or inactivity, leading to reduced muscle strength and an impaired balance with postural sway.

Our data showed that 31.2% of patients had a history of falls; this high prevalence of falls may be explained by a low physical activity in our population, a high prevalence of hypovitaminosis D [28] and irregular grounds in Morocco.

We found a positive correlation between fall and the scores of three tests. It has been shown in many studies that there was a relationship among the older people between balance impairment and a history of falling [29]. Poor physical performance, such as walking speed, lower extremity performance, and balance, increases the likelihood of falling [30]. According to Shumway-Cook et al, [31] the TGUG is a sensitive and specific measure for discriminating between fallers and non-fallers in community-dwelling adults. In contrast, Aslan et al [32] did not find any difference between the scores of the timed balance tests, including the TGUG and STST, while comparing the fallers and non-fallers amongst older subjects. There is a possibility that, even before the fracture, reduced walking speed and balance may partly explain the results of the walking test, as well as the cause of the fracture. Another explanation for slow walking amongst subjects in general is the fear of falling. Fear of falling due to an earlier fall is common, and often results in limited mobility and slower walking speed.

This study presents a number of methodological limitations to be considered in interpreting the results. Since this was a cross-sectional survey, the results must be interpreted carefully. Cross-sectional studies such as this one, can detect associations between variables, but cannot demonstrate causality. A longitudinal study, involving a large cohort, examining the effect of physical performance on bone density, fall and the risk of peripheral fracture, is needed in order to be able to reach generalizable conclusions confidently. Furthermore, one has to be aware of the drawbacks of retrospective fracture and fall registration. However, this study had a number of strengths. Firstly, the study consisted of a large sample size. Secondly, we evaluated three criteria in the same study: bone mineral density; the risk of peripheral fracture; and fall. Another strength was the use of a variety of validated physical performance measures.

## Conclusion

Our data showed that low physical performance is associated with low BMD, and a high risk of falls and fractures. Poorer physical performance was associated with the risk of peripheral fractures in postmenopausal women, independently of bone mineral density. Accordingly, it is recommended that intervention strategies to reduce the incidence of fracture should be targeted at improving both physical performance and bone density by doing regular "weight bearing" exercise.

## Competing interests

The authors declare that they have no competing interests.

## Authors' contributions

HK participated in study design and drafted the manuscript. FA conceived the original idea for the study, supervised its design, performed the statistical analysis and gave critical comments on the draft manuscript. LB enrolled patients, participated in data acquisition and critical revision of the manuscript. LI enrolled patients, participated in data acquisition and critical revision of the manuscript. LE enrolled patients, participated in data acquisition and critical revision of the manuscript. FEA enrolled patients, participated in data acquisition and critical revision of the manuscript. RA conceived the study and performed the statistical analysis. NHH participated in the study design, coordinated the study and gave critical comments on the draft manuscript. All authors read and approved the final manuscript.

## Pre-publication history

The pre-publication history for this paper can be accessed here:


